# Quantitative proteomic analysis reveals key proteins involved in radiation-induced brain injury

**DOI:** 10.1371/journal.pone.0337608

**Published:** 2025-11-25

**Authors:** Jing Liu, Junshuang Wang, Shuang Lv, Hengjiao Wang, Defu Yang, Ying Zhang, Ying Li, Huiling Qu, Ying Xu, Ying Yan

**Affiliations:** 1 Department of Radiation Oncology, General Hospital of Northern Theater Command, Shenyang, China; 2 Graduate School of Dalian Medical University, Dalian, China; 3 Department of Abdominal Radiation Oncology Ward I, Cancer Hospital of Dalian University of Technology, Shenyang, China; 4 Shandong Province Heze Municipal Hospital, Heze, China; 5 Department of Neurology, General Hospital of Northern Theater Command, Shenyang, China; CCMB: Centre for Cellular and Molecular Biology CSIR, INDIA

## Abstract

**Objective:**

Radiation-induced brain injury (RIBI) is a significant complication following radiotherapy for brain tumors, leading to neurocognitive deficits and other neurological impairments. This study aims to identify potential biomarkers and therapeutic targets for RIBI by utilizing advanced proteomic techniques to explore the molecular mechanisms underlying RIBI.

**Methods:**

A rat model of RIBI was established and subjected to whole-brain irradiation (30 Gy). Tandem mass tagging (TMT)-based quantitative proteomics, combined with high-resolution mass spectrometry, was used to identify differentially expressed proteins (DEPs) in the brain tissues of irradiated rats. Gene Ontology (GO) enrichment and Kyoto Encyclopedia of Genes and Genomes (KEGG) pathway analyses were conducted to identify the biological processes and pathways involved. Protein-protein interaction (PPI) networks were constructed to identify key hub proteins.

**Results:**

A total of 35 DEPs were identified, including PHLDA3, APOE and CPE. GO enrichment analysis revealed that the DEPs were mainly involved in lipid transport, cell adhesion, and metabolic processes. KEGG analysis highlighted the enrichment of pathways related to metabolism, tight junctions, and PPAR signaling. APOE was identified as a key hub protein through PPI network analysis, indicating its potential role in RIBI pathophysiology. Immunohistochemistry further validated the increased expression of PHLDA3, APOE, and CPE in the brain tissue of irradiated rats.

**Conclusion:**

This study provides valuable insights into the molecular mechanisms of RIBI by identifying key proteins and their associated pathways. The findings suggest that these proteins, particularly APOE and PHLDA3, could serve as potential biomarkers and therapeutic targets for clinical intervention in RIBI. These results not only enhance our understanding of RIBI’s molecular pathology but also open new avenues for the development of targeted therapies to mitigate radiation-induced neurotoxicity.

## Introduction

Radiotherapy has long been a cornerstone treatment for patients with central nervous system (CNS) tumors, including both primary brain tumors and brain metastases [1,2]. While it effectively targets and eliminates tumor cells, radiation can also cause collateral damage to healthy brain tissue, resulting in RIBI. RIBI can be classified based on the temporal characteristics of clinical manifestations into three phases: acute, early delayed, and late response [3].Pathological features of RIBI include brain tissue necrosis, edema, demyelination, and cognitive and memory impairments [4].These irreversible damages significantly affect patient quality of life and restrict therapeutic options.

The molecular mechanisms underlying RIBI are highly complex, with immune cells playing a pivotal role in both the initiation and progression of brain damage, as highlighted in the literature [5]. The mechanisms underlying RIBI encompass neurogenesis decline, neuronal and glial cell damage, vascular injury, oxidative stress and DNA damage, cell death, and inflammatory responses [6]. Animal model studies have illuminated the involvement of neuroinflammation and identified the simultaneous occurrence of several biological processes, such as disruption of the neurovascular unit leading to blood-brain barrier damage, neuronal progenitor cell death, hippocampal neuroinhibition, and glial cell activation, which contributes to the formation of an aging-associated secretory phenotype [7]. Among these glial cells, astrocytes play a crucial role in maintaining brain homeostasis and function. Their pathological alterations are considered key factors in RIBI pathology and potential therapeutic targets [8]. Therefore, the systematic analysis of RIBI-specific biomarkers and their associated regulatory networks is of significant clinical importance for early injury detection, the development of targeted therapeutic interventions, and the improvement of patient prognosis.

With the advancement of systems biology, quantitative and qualitative proteomics have emerged as crucial methodologies for unraveling complex biological processes. Proteomics has been widely employed to uncover the pharmacological effects, mechanisms of action, and protein targets of natural compounds in cancer therapy [9], as well as to identify abnormalities in disease-related signaling pathways and key regulatory nodes. Mass spectrometry coupled with TMT proteomics provides a reliable approach for quantitative analysis of proteomes [10]. TMT has become particularly popular for disease biomarker discovery, drug target identification, and pathological mechanism exploration due to its high sensitivity, reproducibility, and capacity for parallel analysis of multiple samples [11–13].

In light of this, to investigate specific protein biomarkers of RIBI, we employed liquid chromatography-tandem mass spectrometry (LC-MS/MS) in conjunction with TMT for a comprehensive proteomic analysis of brain tissues from a rat model of RIBI. The goal was to uncover key molecular events associated with RIBI by screening DEPs and analyzing their functional roles, thereby providing insights into the pathological mechanisms of RIBI. Through the identification and functional analysis of DEPs, we aim to elucidate the molecular underpinnings of RIBI, establish a theoretical framework for understanding its pathophysiology, and lay the foundation for future clinical translational research.

## Materials and methods

### Establishment of radiation-induced brain injury model

Male Sprague-Dawley (SD) rats, aged 6–8 weeks and weighing 220 ± 10 g, were randomly assigned to either the control group or the irradiation group. All experimental procedures were approved by the General Hospital of Northern Theater Command Animal Care Committee and conducted in strict compliance with Institutional Animal Care and Use protocols. All rats were housed in groups at room temperature (24 ± 2°C) under a 12-hour light/12-hour dark cycle, with free access to water and food. Rats were anaesthetised via intraperitoneal injection of sodium pentobarbital to minimise discomfort during the irradiation process. Prior to irradiation, the rats were positioned under a CT simulator, and images were uploaded to a treatment planning system to delineate the target area. Whole-brain irradiation was performed using 6 MV X-rays, delivered at a dose rate of 300 cGy/min, with a total radiation dose of 30 Gy. Following irradiation, we monitored animal behaviour such as body weight and locomotor ability, while ensuring adequate nutrition and hydration. On days 3 and 7 post-irradiation, the rat were euthanized by intraperitoneal injection of 150 mg/kg pentobarbital sodium. The death is confirmed by no respiration, pulse, heartbeat and no nerve reflexes for more than 5 min. All necessary efforts were made to minimize the suffering. Brain tissue samples were collected for further analysis.

### Hematoxylin and eosin (HE) staining

Brain tissues from the rats were harvested, fixed in formalin, dehydrated, embedded in paraffin, and sectioned into 5 µm thick slices. The tissue sections were dewaxed, washed, stained with hematoxylin and eosin, and mounted with neutral gum. The stained sections were observed under a light microscope for histopathological examination.

### Protein extraction

Brain tissue samples were homogenized in ice-cold protein lysis buffer and subjected to sonication for 5 minutes in an ice-water bath to ensure complete cell lysis. After centrifugation at low temperature, the supernatant was collected. Dithiothreitol (DTT) was added to the supernatant, and the sample was incubated at 56°C for 1 hour. Iodoacetamide (IAM) was then added to the sample, which was incubated in the dark at room temperature for 1 hour. The sample was precipitated with pre-cooled acetone at −20°C for at least 2 hours, followed by centrifugation. The protein pellet was washed with pre-cooled acetone, resuspended, and protein solubilization was performed with a proteolytic solution.

### TMT labeling

Proteins were digested with trypsin in TEAB buffer at 37°C for 4 hours. Subsequently, an additional trypsin digestion with CaCl₂ was performed overnight. Formic acid was added to adjust the pH to <3, and the sample was centrifuged at room temperature. The supernatant was passed through a C18 desalting column, washed three times, and the eluent was collected and lyophilized. The lyophilized peptides were resuspended in TEAB buffer, followed by the addition of TMT labeling reagent dissolved in acetonitrile. The reaction was incubated at room temperature for 2 hours, and the reaction was quenched with 8% ammonia. Equal volumes of labeled samples were mixed, desalted, and lyophilized for further analysis.

### Fractionation

Mobile phase A (2% acetonitrile, 98% water, adjusted to pH 10 with ammonia) and mobile phase B (98% acetonitrile, 2% water) were prepared. The lyophilized peptides were dissolved in mobile phase A and subjected to liquid chromatography using a Waters BEH C18 column (4.6 × 250 mm, 5 μm) on an L-3000 HPLC system. The column was heated to 45°C, and peptides were eluted in a gradient. Fractions were collected every minute, pooled into 10 fractions, lyophilized, and re-dissolved in 0.1% formic acid.

### Liquid chromatography and mass spectrometry

Mobile phases A (100% water, 0.1% formic acid) and B (80% acetonitrile, 0.1% formic acid) were prepared for liquid chromatography. Each fraction (1 μg) was injected into a nano-scale UHPLC system (EASY-nLCTM 1200), using a homemade pre-column (4.5 cm × 75 μm, 3 μm) and an analytical column (15 cm × 150 μm, 1.9 μm). The Q Exactive™ HF-X mass spectrometer, equipped with a Nanospray Flex™ (ESI) ion source, was employed to generate raw data for subsequent analysis under optimized mass spectrometry conditions.

### Data analysis

The mass spectrometry data were analyzed using Proteome Discoverer 2.4 (Thermo), with a precursor ion mass tolerance of 10 ppm and a fragment ion mass tolerance of 0.02 Da. Fixed modifications included cysteine alkylation, and variable modifications included methionine oxidation, TMT tagging on the peptide side chain and N-terminus, acetylation, and acetylation + methionine loss. The data analysis allowed for the identification of peptides with up to two missed cleavage sites.

### Bioinformatics analysis

Structural domain annotation was performed using InterProScan software, and subcellular localization was determined using CELLO software. DEPs were subjected to Gene Ontology (GO) annotation, Kyoto Encyclopedia of Genes and Genomes (KEGG) pathway enrichment analysis, and protein interaction network analysis. The STRING database was used to construct protein interaction networks, which were visualized using Cytoscape software. Core proteins were identified using the maximal clique centrality (MCC) algorithm. The top 10 core proteins were further analyzed using GeneMania for functional association prediction, integrating co-expression, physical interaction, and pathway co-localization data.

### Immunohistochemistry

Paraffin-embedded brain tissue sections were deparaffinized, rehydrated, and subjected to antigen retrieval. Endogenous peroxidase activity was blocked with hydrogen peroxide, and non-specific binding was blocked with goat serum. Sections were incubated overnight at 4°C with appropriate primary antibodies (PHLDA3, HUABIO, China; APOE, UpingBio, China; CPE, UpingBio, China), followed by incubation with secondary antibodies. Immunoreactivity was visualized using a standard chromogenic detection system, and slides were examined under a light microscope.

### Statistical analysis

Data analysis and visualization were conducted using GraphPad Prism 9.0 software. Comparison of means between two groups was performed utilizing student’s t-test, and statistical significance was considered at *P* < 0.05. (*P < 0.05, ** *P* < 0.01 and *** *P* < 0.001).

## Results

### HE Staining

**Fig 1:**
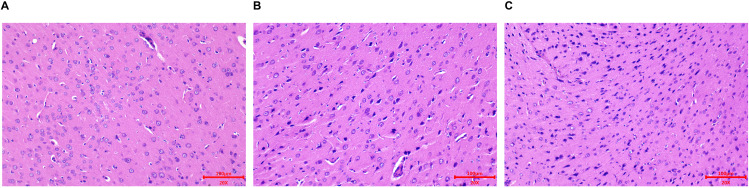
Representative HE staining images of brain tissues. **(A)** Control group. **(B)** 3 days post-irradiation. **(C)** 7 days post-irradiation.

In the control group, the neuronal cell structure appeared intact, with clearly visible nucleoli and a well-organized cellular arrangement. Pathological analysis of brain tissue on the 3rd day after irradiation (30 Gy) revealed signs of cell degeneration and necrosis, characterized by shrunken and lysed nuclei. On day 7 post-irradiation, significant neuronal cell deformation was observed, with a chaotic cellular arrangement. The majority of cells exhibited degeneration and necrosis, with nuclei exhibiting pronounced shrinkage and the presence of cellular vacuoles, as depicted in Fig 1. These findings confirm the successful establishment of the RIBI model.

### Protein Identification

Based on the observed changes in HE staining of rat brain tissue, the 30 Gy group was selected for proteomic analysis. To enhance the quality and reliability of the results while minimizing false positives, Proteome Discoverer software was used to filter the data. Only peptide spectrum matches (PSMs) with a confidence level of ≥99% were considered reliable, and proteins containing at least one unique peptide were included in the final analysis. After performing false discovery rate (FDR) validation, peptides and proteins with an FDR greater than 1% were excluded. A total of 5,583 proteins and 48,378 peptides were identified.

### Principal component analysis (PCA) and repeatability analysis

**Fig 2:**
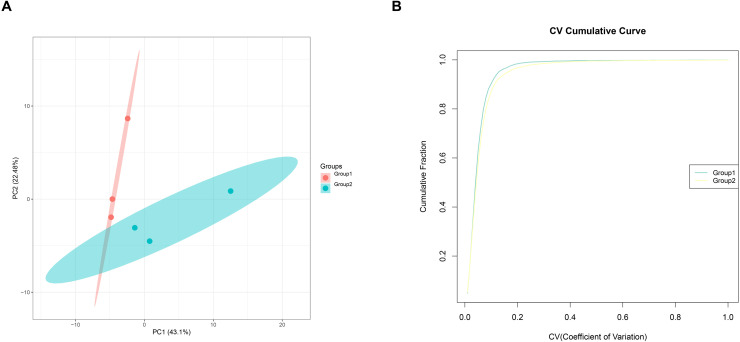
Principal component analysis and repeatability analysis. **(A)** PCA plot. **(B)** Repeatability analysis with CV.

Principal component analysis (PCA) and repeatability analysis were conducted on the data from both the control and RIBI groups. The PCA results, shown in Fig 2, reveal the distinct separation between the control and irradiated groups. The coefficient of variation (CV) further indicated that the sample reproducibility was high, as evidenced by the rapid rise in the curve and the relatively low CV value, suggesting minimal data dispersion.

### Identification of DEPs

**Fig 3:**
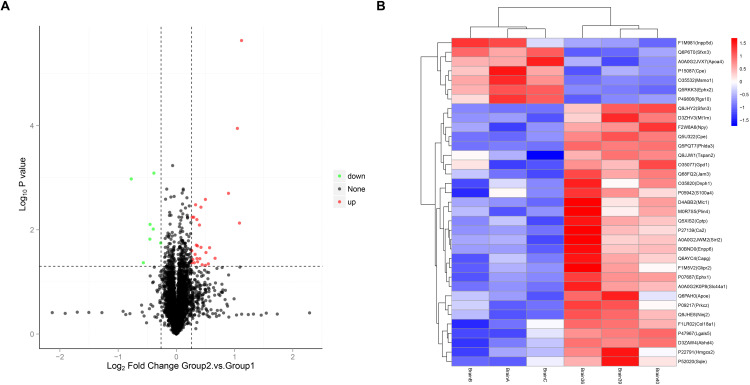
Volcano plot and heatmap of DEPs between RIBI and control tissues. **(A)** Volcano plot of DEPs. **(B)** Heatmap of DEPs.

DEPs were identified based on fold-change (FC) criteria: proteins with FC ≥ 1.2 and P-value ≤ 0.05 were classified as upregulated, while those with FC ≤ 0.83 and P-value ≤ 0.05 were considered downregulated. A total of 35 DEPs were identified, including 28 upregulated and 7 downregulated proteins. The volcano plot and heatmap of these DEPs are presented in Fig 3.

### Subcellular localization and structural domain analysis

**Fig 4:**
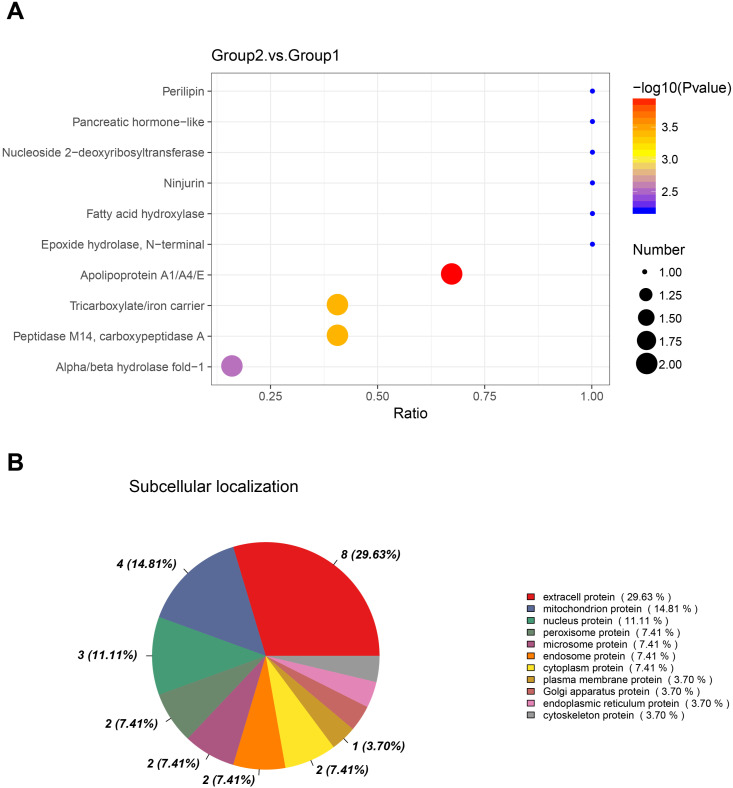
Classification of DEPs based on structural domains and subcellular localization. **(A)** Structural domain analysis of DEPs. **(B)** Subcellular distribution of DEPs.

To further elucidate the localization and functions of the identified DEPs, structural domain and subcellular localization analyses were performed. Structural domain analysis revealed significant enrichment of apolipoproteins A1/A4/E (Fig 4A). Subcellular localization analysis showed that extracellular proteins were the most abundant, as illustrated in Fig 4B.

### GO and KEGG enrichment analysis

**Fig 5:**
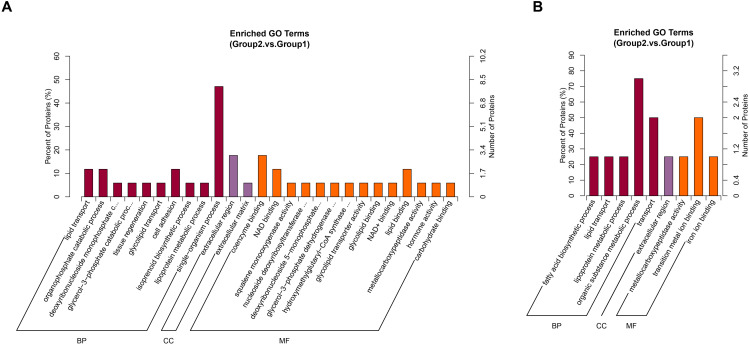
GO enrichment analysis of DEPs. **(A)** GO analysis of upregulated DEPs. **(B)** GO analysis of downregulated DEPs.

**Fig 6:**
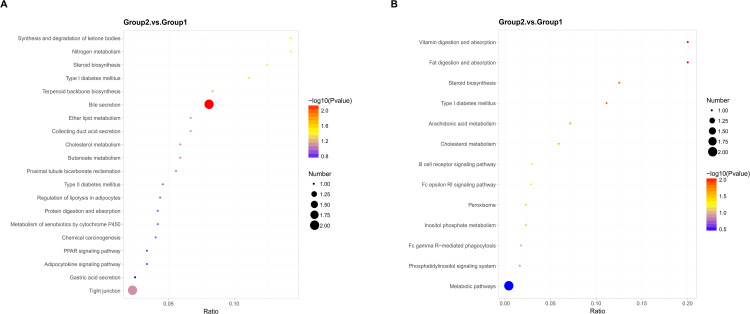
KEGG enrichment analysis of DEPs. **(A)** KEGG analysis of upregulated DEPs. **(B)** KEGG analysis of downregulated DEPs.

GO and KEGG pathway enrichment analyses of the DEPs were performed. Upregulated DEPs were predominantly enriched in biological processes (BP) such as single-organism processes, lipid transport, and cell adhesion. Cellular component (CC) analysis indicated significant enrichment in the extracellular region, while molecular function (MF) analysis revealed enrichment in coenzyme binding and NAD binding (Fig 5). Downregulated DEPs were primarily enriched in organic substance metabolic processes, transport, and metal ion binding. KEGG pathway analysis further showed that upregulated proteins were mainly involved in bile secretion, tight junctions, and PPAR signaling, while downregulated proteins were linked to metabolic pathways (Fig 6).

### Protein-protein interaction (PPI) network

**Fig 7:**
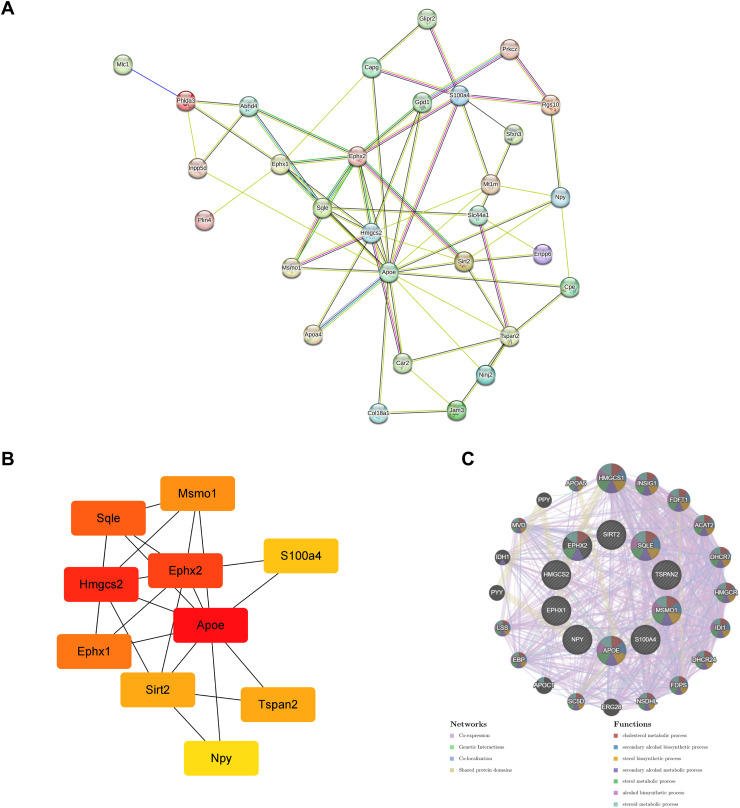
PPI network analysis of DEPs. **(A)** PPI network of DEPs. **(B)** PPI network of DEPs identified by MCC algorithm. **(C)** GeneMANIA analysis of the top 10 hub proteins.

To explore the relationships between the DEPs, a PPI network was constructed using the STRING database and visualized using Cytoscape. The maximal clique centrality (MCC) algorithm was applied to identify the top 10 core proteins, with APOE having the highest score. Further analysis of these core proteins was conducted using GeneMANIA to examine their co-expression, physical interactions, co-localization, and involvement in pathways (Fig 7).

### Immunohistochemical staining

**Fig 8:**
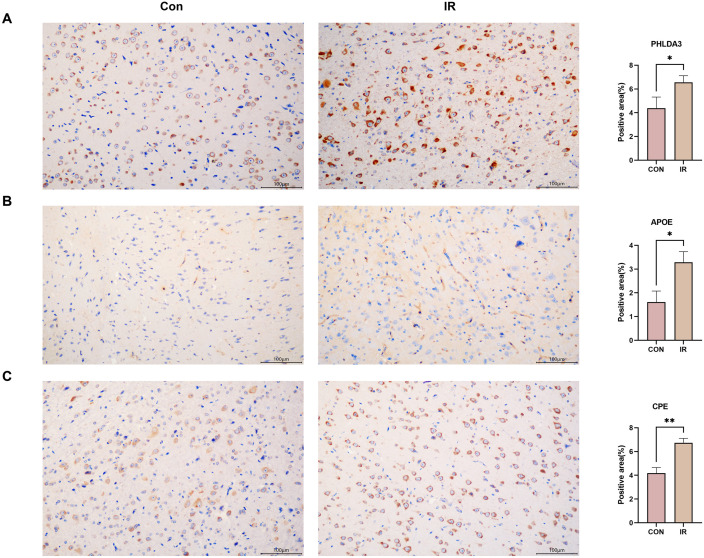
Immunohistochemical staining for PHLDA3, APOE, and CPE in irradiated brain tissues.

Immunohistochemistry was performed to validate the expression of key differential proteins in brain tissue following radiation. The results showed that ionizing radiation significantly increased the expression of PHLDA3, APOE, and CPE in the irradiated brain tissues, as depicted in Fig 8.

## Discussion

Brain metastases are a leading cause of morbidity and mortality in cancer patients [14]. Radiotherapy is an effective non-surgical treatment option for primary brain tumors and their metastases. However, high doses of ionizing radiation, while effective in tumor control, can cause significant damage to brain parenchyma, leading to RIBI [15]. Both brain tumors and their treatments are known to contribute to neurocognitive deficits, affecting domains such as learning, memory, processing speed, attention, and executive function [16]. Clinical studies have confirmed that these cognitive impairments occur across various domains [17]. The mechanisms underlying RIBI are complex, involving a combination of factors such as chronic neuroinflammation, disruption of the blood-brain barrier, oxidative stress, neuronal damage, and exosome secretion-mediated responses [3]. Due to the limited understanding of RIBI mechanisms and the lack of effective preventive or therapeutic strategies, research into potential biomarkers and molecular pathways remains crucial [18].

In this study, we systematically identified 35 differentially expressed proteins, including PHLDA3 and MT1M, using a rat model of radiation-induced brain injury, coupled with TMT-tagged quantitative proteomics and high-resolution mass spectrometry. Our results indicate that these proteins may play significant roles in the pathogenesis of radiation brain injury. GO enrichment analysis revealed that the differentially expressed proteins were primarily involved in processes such as single-organism processes, lipid transport, and cell adhesion, among other biological functions. Further KEGG pathway analysis revealed that these proteins were predominantly enriched in metabolic pathways, tight junctions, and the PPAR signaling pathway. Notably, APOE emerged as the core hub gene through protein interaction network analysis, highlighting its potential role in RIBI.

PHLDA3 is a multifunctional protein involved in a wide range of physiological processes and diseases [19]. It can be induced by various stressors and regulates cellular damage. PHLDA3 plays key roles in vascular development, cellular reprogramming, apoptosis, and oxidative stress [20,21]. Previous studies have linked low expression of PHLDA3 to poor prognosis in esophageal squamous cell carcinoma [22]. Moreover, PHLDA3 has been shown to exert anti-tumor effects in prostate cancer by downregulating the Wnt/β-catenin pathway through inhibition of Akt [23]. Additionally, PHLDA3 inhibition protects against myocardial ischemia/reperfusion injury by attenuating oxidative stress and inflammation via the Akt/Nrf2 axis [24]. In liver injury models, overexpression of PHLDA3 accelerates liver damage through the IRE1-Xbp1s pathway [25]. A recent study indicates that PHLDA3 expression is significantly upregulated in astrocytes derived from amyotrophic lateral sclerosis (ALS) patients, where it promotes cellular stress responses by increasing reactive oxygen species (ROS) concentrations [26]. Our findings suggest that PHLDA3 may influence RIBI through similar mechanisms of oxidative stress and apoptosis, making it a potential biomarker for early diagnosis and a target for therapeutic intervention.

The identification of APOE, the central gene in the protein interaction network, provides new insights into the understanding of radiation neurotoxicity. APOE is a 299-amino-acid protein encoded by the APOE gene, with three isoforms: APOE2, APOE3, and APOE4, each with distinct structural and functional properties [27]. APOE is primarily produced by astrocytes, activated microglia, vascular endothelial cells, choroid plexus cells, and, to a lesser extent, stressed neurons in response to excitotoxic injury [28]. Increasing evidence indicates that astrocytes are the primary producers of APOE in the brain, with this astrocytic APOE being a key driver of pathology. In Alzheimer’s disease models, removal of astrocytic APOE3 or APOE4 leads to a marked reduction in amyloid-β plaque deposition and diminished microglial activation [29]. Astrocytic APOE4 further disrupts cerebral lipid homeostasis by reducing the efficiency of fatty acid transfer from neurons to astrocytes and diminishing fatty acid oxidation within the astrocytes themselves [30]. APOE regulates neuroinflammation through multiple pathways, and APOE knockdown has been shown to induce microglia-associated inflammatory responses in the neonatal mouse brain via astrocytes [31]. Studies comparing irradiation effects in wild-type (WT) and APOE knockout mice suggest that APOE plays a critical role in hippocampal homeostasis and in mediating responses to both acute and chronic low-dose irradiation [32]. Radiation exposure has also been linked to age-dependent cognitive decline and a higher risk of neurodegenerative diseases such as Alzheimer’s disease, in a gender- and APOE isoform-dependent manner [33]. Furthermore, APOE4 has been associated with poor recovery and adverse functional outcomes following traumatic brain injury, with inflammatory and neuronal damage responses observed post-injury [34]. These findings indicate that APOE isoforms may serve as potential biomarkers for risk stratification and personalized treatment strategies in RIBI. Based on these findings, a key direction for our future research will be to selectively overexpress APOE in the brain using transgenic models or viral vector-mediated gene delivery. This will determine whether APOE overexpression increases the risk and severity of RIBI, thereby functionally validating its critical role.

KEGG pathway analysis in this study suggested that metabolic pathways play a significant role in radiation-induced brain injury. A study by Ma et al. [35] demonstrated that patients with acute radiation enteritis, particularly in cervical cancer, exhibited altered metabolic pathways, with specific metabolites identified as potential predictors of radiation enteritis. Furthermore, Ganoderma lucidum polysaccharides have been shown to exert radioprotective effects by enhancing metabolic pathways in serum from radiation-induced mice [36]. Exposure to ionizing radiation disrupts metabolic pathways and induces oxidative stress, leading to organ damage, as shown by urinary metabolomic analysis of mice exposed to high- and low-dose whole-body and localized irradiation, where significant metabolic changes, particularly in the oxidative stress pathway, were observed, with metabolites identified by machine learning models serving as predictors of radiation exposure and biomarkers for radiation-induced damage [37].These findings suggest that ionizing radiation disrupts metabolic pathways and induces oxidative stress, which contributes to brain injury. Our data further support this concept, showing significant enrichment of metabolic pathways in RIBI, indicating the importance of metabolic regulation in radiation-induced neurotoxicity.

Tight junction proteins are crucial for maintaining the structural integrity and function of tight junctions, which are essential in the blood-brain barrier (BBB) [38]. Whole-brain irradiation has been shown to induce damage to tight junctions [39]. Notably, it has been proposed that targeting the deubiquitinase USP11 may mitigate radiation-induced pulmonary fibrosis by modulating endothelial tight junctions [40]. The tight junctions in the BBB play a critical role in protecting the brain from harmful substances. Following brain injury, inflammatory mediators such as cytokines, chemokines, and growth factors are released, disrupting the integrity of intercellular junctions and causing imbalances in cellular polarity, molecular regulation, and inflammatory responses [41]. The small molecule dye IR-780 has been shown to ameliorate cognitive dysfunction, reduce neuroinflammation, and restore tight junction protein expression in the BBB after whole-brain irradiation [42]. Our results further support the critical role of tight junctions in RIBI.

PPAR are ligand-activated transcription factors belonging to the nuclear receptor superfamily. PPARα, in particular, is expressed in tissues responsible for fatty acid catabolism and regulates numerous metabolic pathways [43]. All PPAR isoforms have been implicated in the development and function of the brain [44]. PPAR ligands have shown promise in regulating pro-inflammatory pathways and upregulating antioxidant enzymes, providing an effective therapeutic approach to mitigate the delayed effects of whole-brain radiotherapy. PPARα ligands inhibit radiation-induced pro-inflammatory responses in microglia in vitro and prevent the detrimental effects of irradiation on hippocampal neurogenesis in vivo [45]. PPARδ has been shown to inhibit radiation-induced inflammation in microglia through suppression of the NF-κB and PKCα/MEK1/2/ERK1/2/AP-1 signaling pathways [46]. Our findings of significant enrichment in the PPAR signaling pathway suggest the therapeutic potential of targeting the nuclear receptor network in RIBI.

Our study integrates bioinformatics and experimental validation to identify PHLDA3, APOE, and CPE as key molecules in the RIBI pathogenesis. To enhance the clinical translatability of these findings, we propose a pathophysiological framework categorising these molecules according to their dominant roles within the injury cascade: APOE relates to neuroinflammation and blood-brain barrier integrity, PHLDA3 to oxidative stress and apoptosis, and CPE to blood-brain barrier/tight junction disruption. This conceptual model links our molecular discoveries to clinically recognised core pathological processes, providing a durable and intuitive framework for understanding RIBI pathology. It not only elucidates the biological significance of these biomarkers but also establishes a foundation for future development of pathway-specific diagnostic and therapeutic strategies. In conclusion, this study provides valuable insights into the molecular mechanisms underlying radiation neurotoxicity by systematically screening for RIBI-related differential proteins and analyzing their regulatory networks. However, limitations remain. Firstly, this study was conducted exclusively in male rats. This choice was primarily made to eliminate potential confounding effects of the oestrous cycle and sex hormones on neuroinflammation and repair processes following brain injury. While this design enhances internal consistency, it limits the generalisability of our findings to female subjects. Future investigations should explicitly include both sexes to examine potential gender-dependent variations within the RIBI molecular machinery, which is crucial for developing personalised therapeutic strategies. Secondly, further studies using in vitro cell models are needed to confirm the biological functions of these key proteins. Additionally, a multi-omics approaches to explore the spatial and temporal dynamics of these molecules and their interactions in greater detail.

## Conclusion

In this study, TMT-based quantitative proteomics was employed to systematically identify DEPs in RIBI, along with their enriched pathways and protein interaction networks. These findings suggest that these proteins may serve as potential therapeutic targets for RIBI. By enhancing our understanding of the molecular pathology of RIBI, this research provides valuable insights into potential clinical intervention strategies.

## Supporting information

S1 Table Differentially expressed proteins. (XLS)

S2 Table DEPs based on structural domains. (XLS)

S3 Table DEPs based on subcellular localization. (XLS)

S4 Table GO analysis of upregulated DEPs. (XLS)

S5 Table GO analysis of downregulated DEPs. (XLS)

S6 Table KEGG analysis of upregulated DEPs. (XLS)

S7 Table KEGG analysis of downregulated DEPs. (XLS)
